# Power system transformation in emerging countries: A SWOT/PESTLE analysis approach towards resiliency and reliability

**DOI:** 10.1016/j.heliyon.2024.e33018

**Published:** 2024-06-14

**Authors:** Kokou Amega, Yacouba Moumouni, Yendoubé Laré, Ramchandra Bhandari, Pidename Takouda, Saidou Madougou

**Affiliations:** a West African Science Service Centre on Climate Change and Adapted Land Use (Wascal), University Abdou Moumouni of Niamey, Po. Box:10662 FAST/UAM, Niamey, Niger; b Higher Colleges of Technology, Electrical and Electronics Engineering, Ras Al Khaimah Women's Campus, Po. Box: 4792, Ras Al Khaimah, United Arab Emirates; c University of Lomé, Department of Physics, Centre d’Excellence Régional pour la Maîtrise de l’Electricité, Po. Box:1515, Lomé, Togo; d Institute for Technology and Resources Management in the Tropics and Subtropics (ITT), Technische Hochschule Köln, Betzdorfer Strasse 2, 50679, Cologne, Germany; e Direction Régionale Maritime de la CEET, 426 Avenu Maman Fousseni, Po. Box: 42, Lomé, Togo; f Laboratory of Energetics, Electronics, Electrical Engineering, Automation and Industrial Computing, University Abdou Moumouni of Niamey, Niger, Po. Box: 10963, Niamey

**Keywords:** SWOT/PESTLE, Power system, Transformation, Reliability, Resiliency, Togo

## Abstract

Most developing countries' electric power system is stressed by an unprecedented demand growth as well as obstacles that call for urgent actions. Therefore, tackling the present-day power-related challenges and ensure dependable and safe electricity may result in improving living conditions. This research aims to comprehend the primary factors that impede power companies in emerging economies and propose ways of addressing them with a focus on Togolese electricity system as a case study., The methodology utilized to study a complex and dynamic system like electricity sector is an integrated model composed of a survey and review of available literature, an interview with energy experts and the SWOT/PESTLE analysis to perform an in-depth and all-encompassing analysis. The study revealed that the electrification poverty was 39.47 % at countrywide level that requires an additional power of 220.95 MW to that of 2021 to achieve 100 % of electricity access by 2030. Moreover, the system's performance is hindered by a number of internal and external bottlenecks. They include but not limited to limitations in policies and regulations; technical difficulties in the transmission, distribution and off-grid subsystems; insufficient investments; and a lack of incentives and taxes rebates. In light of these findings, a model prioritizing a resilient power system was proposed for transforming the outdated power infrastructure in developing countries laying stress upon energy mix planning, transmission and distribution subsectors innovation and effective regional collaboration.

## 1 Introduction

Continued economic and population growth drives energy demand at national and global levels [1,2]. As a result, electricity demand is growing at an unprecedented rate in contrast to the growth of grid installations and enlargement of electricity capacity, especially in developing countries (DC). This difference is more pronounced in central energy generation systems than in decentralized energy generation systems [3]. This situation has led to a couple of problems **–** excessive demand, limited access, blackouts, ageing transmission and distribution systems, safety and reliability as well as resilience issues, and extreme climate change **–** that the power sector faces in most DCs these days. Such issues threaten the systems' functionality to grant electricity constantly, withstand injuries and stand reliable and resilient, whilst non-stop provision of electricity is of utmost importance for society [4]. The resilience of an electricity system refers to its capability to recover quickly from any unfavorable impact due to natural hazards that may hamper its functionality. Electricity systems may be reliable in case electric power is continuously furnished to consumers [5]. Thus, reliability is not a function of individual generating systems, but rather a result of how the entire power system works. A reliable and resilient electricity grid constitutes the backbone of strong economic growth and a society's sustainability. To meet such increasing electricity demand, utility corporations are not only diversifying their power generation capacity, but are also increasing it. It remains important to note that the aforementioned generation capacity is mostly fossil-fuel based. Because of the depletion of fossil sources in addition to being accountable for a major part of greenhouse gas (GHG) emissions, decentralized energy systems are nowadays thought to play a key function in supplying the indispensable electricity to balance consumption at all levels [6]. Unfortunately, the design of existing electric power systems frequently does not allow large-scale integration of decentralized renewable resources (DRRs). Furthermore, fossil fuel-based power generation is not only unsustainable, but also extremely harmful to the environment.

In contrast to developed economies, energy systems in emerging economies may be considered highly unsustainable due to lower levels of ethics, less focus on best practices, and scarce resources [7]. In spite of that, it is worth of note that integrating large-scale of renewable energy onto the grid can increase the existing energy balance requirements at all levels of the system [8]. Accordingly, the current electricity system needs to be transformed and redesigned to achieve a high grid penetration rate of distributed renewable energy (DRE). To this end, the improvement of power systems has been of great interest to researchers who have proposed short and long-term strategies to enhance the resilience of the power system. Authors in Ref. [9] demonstrated that combining proactive actions, dynamic topology, a mobile unit allocation that is dispatchable and reconfiguring the distribution feeds result in significant outage management. In Ref. [10], setting demand response programs and implementing mobile energy storage alongside integrating energy resources have enabled resilience improvement. In Ref. [11], the application of techniques, such as 1) electricity supply maximization, 2) changing network topology, and 3) direct load control programs have led to improving distribution network resilience. Likewise, the power system's resilience has been enhanced by integrating various microgrids powered by a gas-to-electricity system (viz., combined heat power technology) to address outages in the main electrical grid [12].

In addition, the installation of a multicarrier coupled with fast switching strategies of the transmission lines led to the power system's improvement in the advent of extreme weather [4]. An ensemble of distribution, generation, storage systems, smart homes, and plug-in electric vehicle schemes have been used to effectively manage uncertainties associated to power demand and renewable energy variabilities, thereby improving the electrical system [13,14]. It has also been underlined the effectiveness of enhancing the resilience of power systems through mobile batteries, hydrogen storage tanks, and fuel cell usage [15]. Moreover, smart buildings equipped with photo voltaic panels and energy storage systems have been used to perform network voltage profile improvement [16].

Based on the above-mentioned state-of-art features, improving the power system is a task that starts with the generation (related to the main grid and microgrids) and passes through transmission, distribution, end-users’ premises, and finally ends with the demand response management (DRM) [[17], [18], [19]]. Hence, power systems’ resilience may be enhanced at all the above stages of the system. Ensuring power system resilience that supplies reliable power would not be too much sacrifice for a nation due to the fact that the economic long-term development and community well-being are at stake. Thus, accomplishing this standard in emerging nations would be a priceless contribution to their sustainable development.

It remains a fact that there is a little research done on energy systems in developing countries that have conducted an in-depth and all-encompassing analysis of the power sector to bring out its real state. Electricity utilities pay dearly for the lack of these kinds of studies which are intended to help them in the maintenance, upgrading and better planning of the sector. Therefore, this study attempted to assess deeply the Togolese electric power system in all aspects as a case study and portrayed its actual picture to bridge the gap. Undoubtedly, leveraging on existing insights and expertise, power system in emerging nations may be audited with corresponding fundamental key points formulation for policymakers to plan better and address weaknesses brought out. Based on a literature review and interviews with energy experts, the methodology provided a comprehensive assessment of the Togolese energy sector, which was then reviewed through the SWOT/PESTLE analysis and a proposal for fundamental changes to improve power quality and reliability abroad had been proposed. Otherwise, one would not get insightful knowledge and a consistent and coherent evaluation of Togo's existing power systems.

The study is useful in the way it reveals up the effort and the accountability of every actor intervening in the energy sector for the way forward. Consequently, the novelty of this research resides in the integrated model utilized. It is a combination of a survey and review of available literature on Togolese electricity system, an energy expert elucidation through interviewing of energy specialists employed in the sector and the SWOT/PESTLE analysis used together to perform the work. Conducted for the first time on the Togolese power system to our knowledge, the present study contributes to the existing literature in a number of ways. In as much as possible, the overall situation of the Togolese electricity system was circumcised under the current political (Decision), economic (Profit), and social (People) environment (Planet) ecosystems. In other words, the study looked into the triple bottom lines of “Sustainability.” Accordingly, this study provided a bird's eye view of the whole system and demonstrates how factors that compose the acronym PESTLE (which stands for political (P), economic (E), social (S), technological (T), legal (L) and environmental (E)) may additionally infer or impede the electricity energy structures. More, the study drew one's attention on the country's electricity poverty and projected the minimal additional power to be provided to achieve 100 % of electricity access by 2030. Moreover, tentative solutions were provided to address issues facing in the sector.

The main objectives of this work are to 1) conduct an in-depth and all-encompassing analysis of electric power system in DCs and thereby 2) determine the system's bottlenecks as well as factors influencing it and 3) propose corresponding solutions. It is worthy of note to point out that the energy sector, in most DCs, is under the control of the state utility company along with issue associated to openness and the power grid is still in traditional configuration (i.e., non-smart). Hence, electrical power is produced and transported at high voltage for long distance and distributed at medium and low voltage to the consumers. Understanding such system is a tough task and, therefore, an integrated approach may be helpful.

The following sections describe the research methodology. Section 3 presents the results and discussion and the policy implementations. Finally, Section IV, briefly, concludes the study.

## 2 Methodology

To fulfill the objectives mentioned above, an integrated model has been utilized. It includes 1) a survey and review of handy literature grabbed from institutions pertaining to the electric power sector, 2) interviews with energy specialists from utility companies, 3) the SWOT/PESTLE analysis approach, and 4) an oriented-based model of a resilient power system. The literature survey/review and interviews with specialists on the existing Togolese electricity system aimed at collecting input data and setting the prerequisite for the SWOT/PESTLE analysis to draw the actual picture. Based on later information, policies were formulated, as suggestions, to transform the system into a more reliable, resilient, and sustainable model. MS Excel has been used to process data and draw the figures.

### 2.1 Literature survey/review and energy experts’ interview

**Fig. 1:**
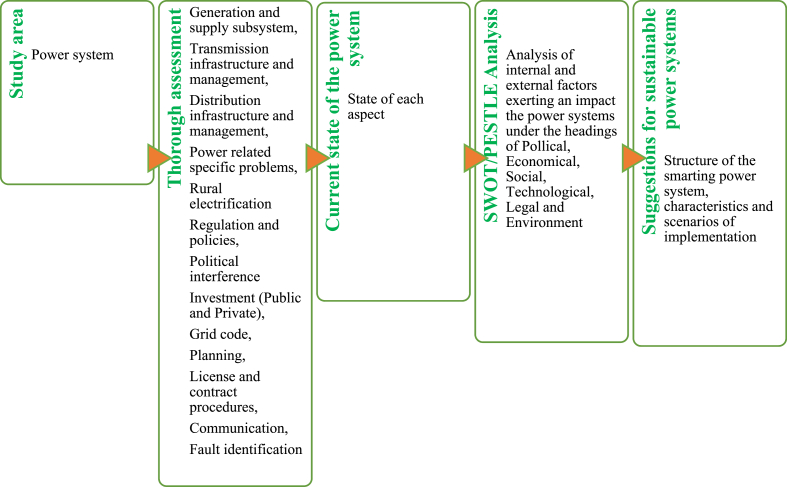
Methodological SWOT/PESTLE-based framework. Source: Authors.

To collect the primary data on Togolese electric power system a survey and review of available literature from 2001 to 2021 and an interview of energy specialists from public institutions and agencies in charge of power sector have been conducted. Existing and available literature consists of studies, annual activities reports produced by the Electricity Sector Regulation Agency (ARSE), the international utility company (Benin and Togo) in charge of the transmission system (CEB), the national utility company of electricity distribution (CEET), and the Togolese Rural Electrification and Renewable Energy Agency (AT2ER), policies, laws and decrees framed by the State and the Ministry in charge of energy. It also includes regional policies and regulation. Table 1 depicts surveyed literature. The ‘x’ in the table stands for ‘the document deals with’. The survey and the review have the function of allowing to harvest information of on the system and its surroundings over 21 years. Furthermore, an insightful interview was conducted with energy specialists and employees from CEET, AT2ER, and the Ministry of Energies to shed more light on the current situation (what is missed in the surveyed literature) and the critical point of view of the power system. The questionnaire used during the interview was a modified questionnaire of the Economic Commission for Africa of the United Union [20]. Various aspects were taken into account, as can be seen in Fig. 1.

**Table 1:**
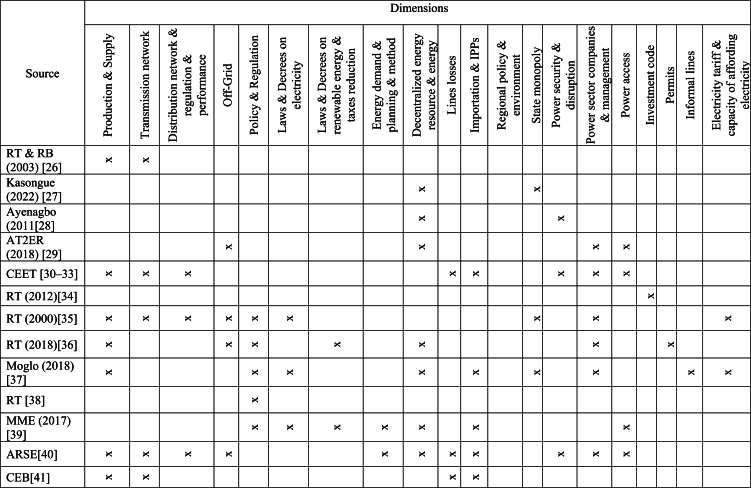

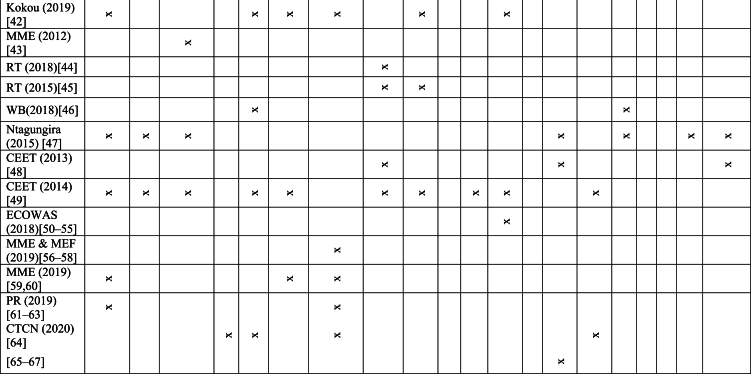
A literature survey [[21], [22], [23], [24], [25], [26], [27], [28], [29], [30], [31], [32], [33], [34], [35], [36], [37], [38], [39], [40], [41], [42], [43], [44], [45], [46], [47], [48], [49], [50], [51], [52], [53], [54], [55], [56], [57], [58], [59], [60], [61], [62]].

### 2.2 SWOT/PESTLE analysis approach

SWOT is the acronym for strengths (S), weaknesses (W), opportunities (O) and threats (T), whereas SWOT analysis is a tool applied to group all internal factors (strengths and weaknesses) and the external factors (opportunities and threats) that may affect a system from the inside and outside [63]. Strengths and weaknesses stand, respectively, for the advantages for an organization from the implementation of its plan and obstacles preventing the successful implementation of the initial goals. Opportunities portray a conducive environment, while threats refer to unfavorable conditions for the system to stand over the years. A disadvantage of using SWOT analysis is that its performance is limited at the description stage and does not go beyond [64]. To overcome this limitation, multiple tools or techniques must be applied for better comprehensiveness rather than one in a system analysis because they provide many strategic benefits to system performance, creativity, and learning [65].

A PESTLE analysis tool, as far as it is concerned, is part of marketing principles used to analyze industries and companies as well as energy sectors considered as system It helps to provide insights into factors that influence systems from different angle and classifies them into political (P), economic (E), social (S), technical (T), legal (L) and environmental (E) factors:

⁃ Political factors are factors that describe the influence of the State that may affect the electricity sector through the energy policies
⁃ Economic factors are factors deal with the possibility of investment in the power sector, the impact of power supply in the economic growth, the purchasing power of end-users, power project financing possibility.
⁃ Social factors concern the willingness, readiness and the reluctance of the population regarding the electricity usage and purchasing.
⁃ Technology factors describes the power sector's infrastructures and equipment available and their impact of the system, system's technical operation, technical expertise and skill of people working in the institution in charge of the sector as well as those institutions and agencies
⁃ Legal factors pertain to the external and internal influence of laws, decrees/orders and standards pertaining to power sector.
⁃ Environmental factors include but not limited to weather conditions, geographical location, environmental offsets that may influence the power sector.

As complementary tool, PESTLE analysis allows further analysis emphasizing on internal and external factors identified by the SWOT tool for more comprehension. Accordingly, SWOT and PESTLE synergy leads to deep analysis and a better understanding of the situational analysis of a system [66,67].

**Fig. 2:**
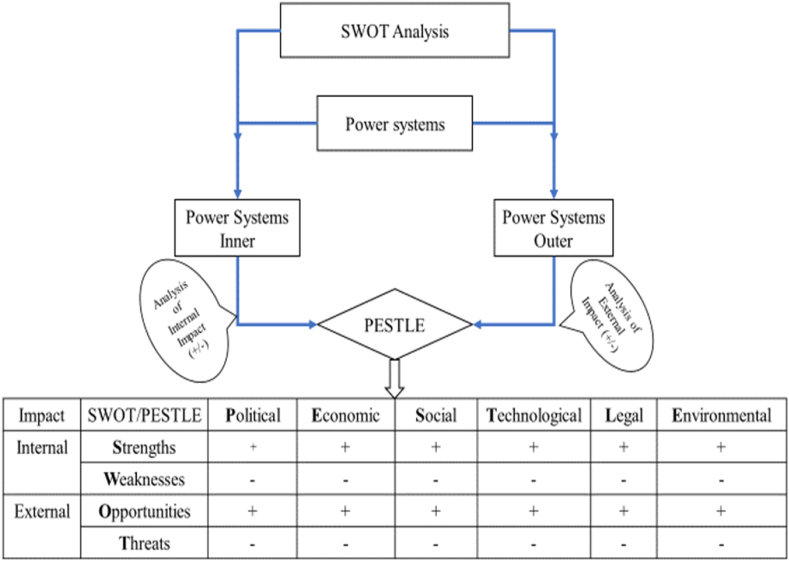
SWOT and PESTLE analysis flowchart. Source: Authors.

This present study combined both tools as presented in Fig. 2 to comprehensively identify as much as possible all factors that impact the power system for better decision-making.

### 2.3 Oriented-based model of a resilient power system

The development of an oriented-based model of a resilient power system was attempted. Such a model seeks to pave the way to attaining the objective of the resilient and reliable electric power system. Hence, a reliable and resilient power system constitutes the backbone of strong economic and sustainable development whether in industrialized and emerging countries. Togo is not an exception. Toward this perspective, this model has been developed to transform the Togolese power sector, considering the country's political and socio-economic context in the short, medium, and long terms, as a response to the plethora of difficulties encountered in the sector. As a result, various scenarios were proposed to guide (or support) the alternatives.

## 3 Results and discussions

### 3.1 Status of the Togolese power system

The electric power system in the study is considered a system consisting of electricity generation, transmission and distribution sub-sectors, demand management and the governance of the whole system. With that understanding, the Togolese electric power system has been assessed. Such assessment has led to a thorough analysis of various aspects of the system presented in the following sub-sections.

#### 3.1.1 Togolese power system management

This section answers the question ‘What are the key actors and who is in charge of the electricity sector in Togo?’. Thus, the Togolese electric power system is a vertically integrated public company and independent power producers (IPPs) [48]. In 2021, the four corporations operating in the existing electricity sector were 1) the international utility company CEB responsible mainly for the transmission subsector, 2) the national utility company CEET responsible for electricity importation, distribution and selling across the country, 3) Contour Global Togo SA (CGT), an independent power producer, and 4) AMEA TOGO SOLAR SAU, also an independent power producer. To sum up, this power sector is under the control of the State.

#### 3.1.2 Power demand and supply

**Fig. 3:**
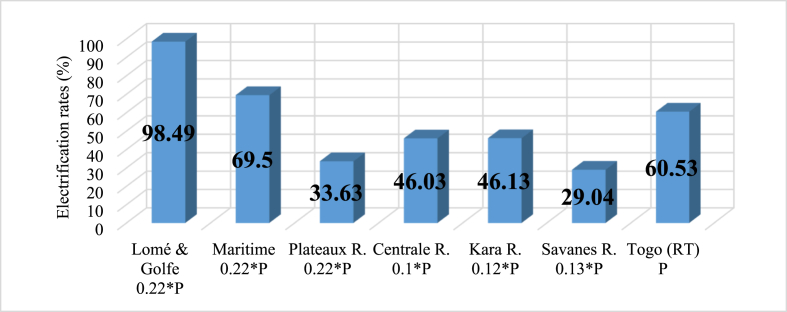
Electricity access rate at regional and national levels in the distribution network in 2021 [35].

**Fig. 4:**
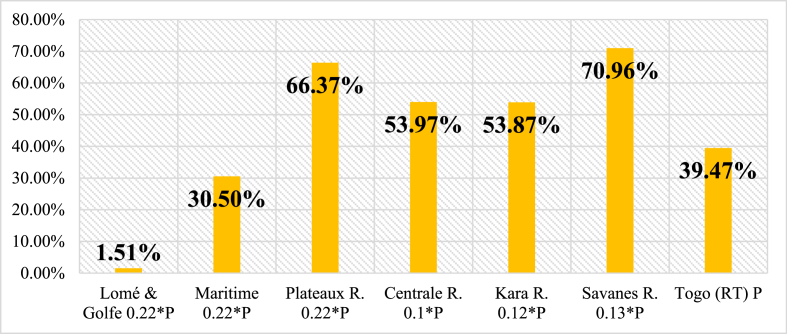
Electrification poverty across the regions.

**Fig. 5:**
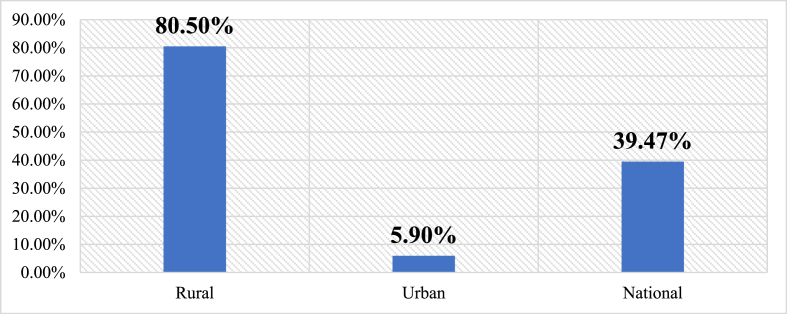
Electrification poverty in rural area vs urban area.

**Fig. 6:**
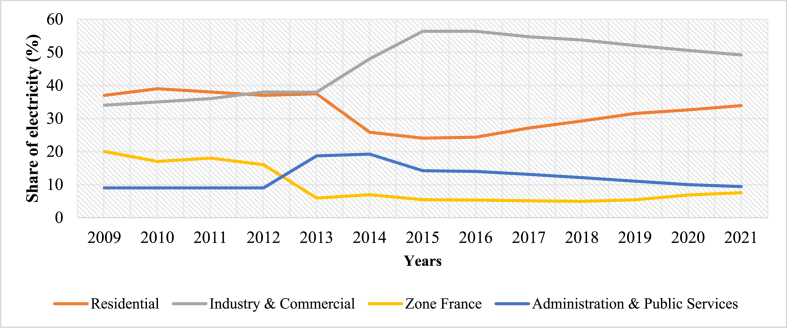
Share of power demand by sectors, Togo [35].

**Fig. 7:**
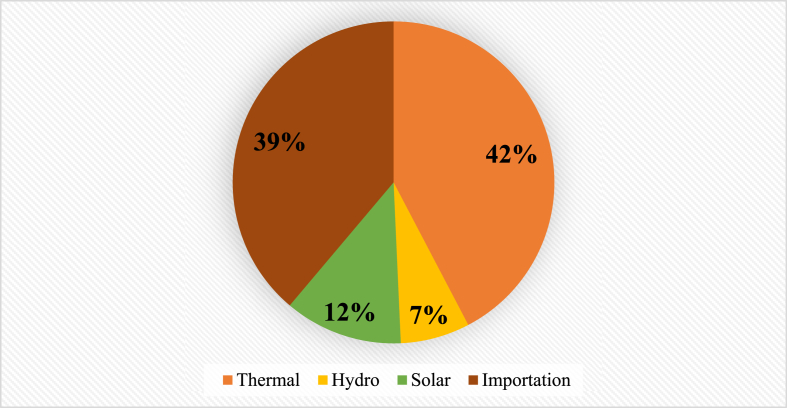
Share of the energy mix in 2021 [35].

This section presents people's demand for electricity whether in residential, commercial, public services and industry and the provision made to satisfy that need. The assessment reveals that Togo lacks enough electrical energy to meet its growing demand, as access to electricity is still limited [68]. Notably, Fig. 3 depicts regional electricity access rates [69]. Considering that figure, it can be seen how power is unevenly distributed across regions. The deficit in electrification in administrative regions as well as in rural and urban areas is portrayed in Fig. 4, Fig. 5. Current power consumption is much more dependent on economic activities and income than on population (P). Thus, Lomé, the capital city of Togo, takes the lead in power demand because it is the most developed region with numerous economic activities. In addition, Fig. 6 presents the shares of national electricity demand by economic sectors. Industry leads in power demand followed by the residential sector. Power supply and demand are closely linked. In 2021, energy supply could not balance demand because power production was not growing as fast as demand. Table 2 shows the available supply capacity across the country and the corresponding companies and sources, while Fig. 7 depicts the status of the energy mix.

**Table 2:** Power supply in 2021 [35].

COMPANIES	REGIONS	CAPACITY (MW)	TYPES
CEET	Maritime	16.5228	Thermal
	Plateaux	0.392	
		1.6	Hydro
		0.15	Solar
	Central	0.4912	Thermal
		0.25	Solar
	Kara	4.88	Thermal
		0.1	Solar
	Savannah	2.358	Thermal
		0.1	Solar
CEB	Plateaux	32.5	Hydro
	Maritime	20	Thermal
CGT		100	
KEKELI Efficient Plant S.A.		47	
AMEA SOLAR TOGO SAU	Central	50	Solar
Importation		190

#### 3.1.3 Power systems infrastructures

**Fig. 8:**
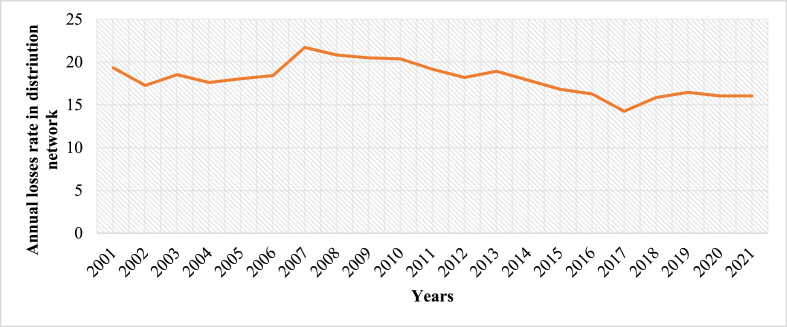
Losses in the distribution network in 2021 [35].

**Fig. 9:**
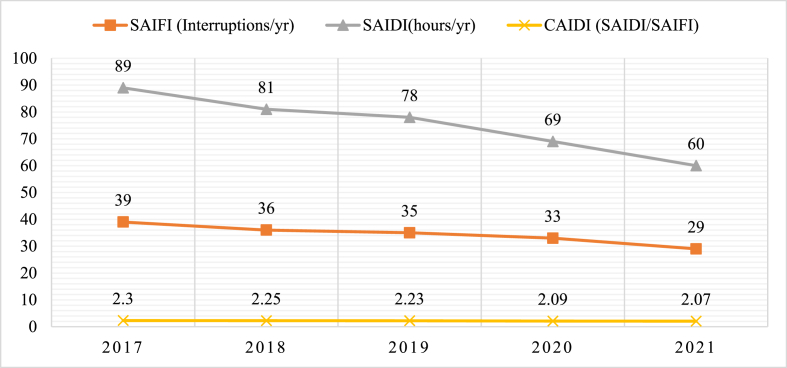
Distribution network performance indicators in Lomé.

This section presents power grid in Togo. It is all about the electricity transport lines found throughout the country that form the transmission and distribution subsystems. Distribution lines are divided into urban, interurban, and rural lines. The transmission lines consists of high voltage like 330 kV, 161 kV and 66 kV whilst distribution system typically consists of medium and low voltages, such as 34.4 kV, 33 kV, 20 kV, 5.5 kV, and 0.4 kV [69]. Transformers at the distribution level step down the lines' voltage to 0.4 kV and 220 V for industrial and residential consumers, respectively. Until 2020, the distribution infrastructure was still limited, even in the largest cities, where there are vast informal lines from grid-connected homes to many quarters. Among the issues encountered in this subsector, it is pertinent to mention line losses as depicted in Fig. 8. This phenomenon is an indication of the need to improve the efficiency of the subsystem. Moreover, power security and reliability need to be improved because of the fact that power interruptions happen year to year. These interruptions take into consideration those related to incidents, scheduled maintenance, blackouts and load shedding recorded on the distribution network for more than five (05) minutes. Interruptions that have occurred during the past five years have been factored in the calculation of performance indicators such as system average interruption frequency index (SAIFI), system average interruption duration index (SAIDI), and customer average interruption duration index (CAIDI) and presented in Fig. 9. It can be observed that interruption regardless of the origin lasted at least 2 h (CAIDI >2 h) in Lomé from 2017 to 2021. At the national level, SAIFI and SAIDI statistics were 1000 interruptions and 755 h, respectively, in 2016, the highest score in the region as reported in Ref. [47]. Conversely, the transmission subsystem consists primarily of high-voltage infrastructures. This power subset is composed of line voltages of 61 kV, 66 kV, and 34.5 kV. In addition, the Togolese high voltage lines terminate at 161 kV/11 kV, 161 kV/20 kV, 161 kV/22 kV, 161 kV/34.4 kV, 161 kV/63 kV, and 161 kV/66 kV stations.

#### 3.1.4 Communication and metering schemes

This section presents how information is conveyed inside the system and means of electricity metering. The traditional means of communication between power operators and consumers (telephone, mail, visits, WhatsApp) remain in use. The power system sector is under a transformation process through the performance agreement between the State and CEET. However, the implemented metering system may prove to be an important step toward modernization. Thus, there are two types of electricity meters. The traditional measurement system (postpaid) in which a CEET technician must regularly return to the clients' premises to record their consumption. The second option is a prepaid electricity metering system. The following are general methods of communicating with the distribution system:

⁃ Technicians at the dispatching stations record the consumption per hour (kWh) and communicate the values to the Central Control Office. A SCADA project is underway and should be operational by 2024.
⁃ The endpoints, i.e., the station and end-user, have the technology to communicate remotely. But there is no supervisory system installed yet. Thus, remote management is not possible to be done at the moment.

In the event of a fault, the breaker at the MV station opens automatically. The faulty section is not automatically detected. A trial and fault search are carried out to identify the faulted portion. Installation of communicating fault detectors would allow for automatic detection, thereby saving time and labor costs.

### 3.2 SWOT/PESTLE analysis of the Togolese power systems

The outcomes of the combination of SWOT and PESTLE tools to analyze the Togolese power system are presented in this section. It presents identified key components of the power system and their analysis under the heading of strengths, weaknesses, opportunities and threats (SWOT) as well as their classification into political, economic, social, technical, legal and environmental (PESTLE) factors as can be observed in Table 3, Table 4. The fundamental picture of the country's electricity system is highlighted by both internal and external factors influencing it. The Togolese power system has various strengths varying from political to environmental. Some key basics are set up and efforts have been made. Due to the key role the energy sector performs in the country, the State is paying special attention to it. A few of those strengths are the political will portrayed in the laws voted and decrees that have been issued, the establishment of regulation and management entities, some success in terms of electrification rate and the number of households having access to the grid. For a continual power supply to meet the increasing population and economic growth, these positive points have to be strengthened and sustained for the system to stand over generations. Nevertheless, weaknesses do exist in the system and they might be pulling in some ways down the efforts made within and for the sector. The performance of the regulation governance is still low. The investment has been limited over the years as such funds for research in renewable energy does not exist. 37.5 % of the population still needs access to electricity mostly in the rural areas where 80 % lack access. The T&D losses (16.4 % in 2021) remain higher than the standard level of 10 %. As far as power outage is concerned, power supply has been disrupted over the years, and therefore, there is a need to improve and update the power system quality and security standards for transmission and distribution. The enabling environment is set by the existing opportunities that may work for the sector (Table 4). The opportunities derived from the policies developed in the regional and international agreement that Togo abides with. Such opportunities may be from economic, technical, social, legal and environmental aspects which the country must to take advantage of on the condition of minimizing and dealing with the surrounding threats wherever they come from.

**Table 3:** SWOT/analysis of internal factors outputs.

SWOT/PESTLE	Internal factors	
	Strengths (+)	Weaknesses (−)
Political	♦ Generation governed by the International agreement on the revised Benin-Togolese electricity code (2003), ♦ Transmission governance as in the International agreement on the revised Benin-Togolese electricity code (2003) ♦ Approval of the Technical Regulations for the Distribution of Electrical Energy in Togo (RTD) ♦ Energy strategy and energy planning policy (generation, transmission, distribution and Off-Grid) as in Electrification strategy (cite) ♦ Power system performance policy ♦ Political will and commitment of the government ♦ Energy policy letter available ♦ Electrification strategy available ♦ Regulatory framework favorable to the development of renewable energies ♦ Tax and duty relief measures on renewable energy project equipment importation ♦ Liberalization of electricity production	♦ Monopoly of the State ♦ Influence of politics in most power system aspects ♦ Regulation governance performance still low (Togo ranked 26th out of 43 countries in 2021) ♦ Poor governance ratio (from −0.85 to −0.75 as per 2010 and 2019 respectively
Economic	♦ Electricity powering economic activities ♦ Power fueling the country's economic development	♦ Limitation of power utility auto-financing ♦ Tariff adjustment is very difficult ♦ The method of electricity price determination is rigid and not necessarily adapted to the economic context ♦ The more consumption, the higher the rate/kWh ♦ Costs of maintenance of infrastructures during operation ♦ Investment renewal and upgrade of the system infrastructure and equipment ♦ Absence of a national fund for the development of renewable energy ♦ Lack of funding for research in the renewable energy sub-sector
Social	♦ 60.53 % of access to electricity in 2021 ♦ Citizens employment ♦ Population well-being and lifestyle improvement ♦ Cooling of hospital equipment and products ♦ Lighting of schools and streets ♦ Improvement in communication ♦ Increase in the number of people connected to the grid over the years ♦ Strong involvement of CSOs and the private sector in the development of RE	♦ 39.47 % lack access to electricity mostly in rural areas where the rate of access was 19.50 %. ♦ Occurrence of power outages and disruption ♦ Costs of subscription to electricity lines still high for a fringe of the population
Technical	♦ Existence of utilities in charge of power production, purchasing and distribution (CEB, CEET) ♦ Existence of the electricity regulation authority (ARSE) ♦ Creation of rural electrification and renewable energy agency (AT2ER) ♦ Existing power systems infrastructures ♦ Existing thermal power plants across the country with different capacities ♦ Existing solar plants (large and mini) ♦ Existence of Grid code for generation, transmission, and distribution ♦ System quality and security standards developed for the generation ♦ Limited production to meet the increasing demand ♦ Declining in transmission and distribution line losses ♦ Availability of human expertise and know-how in the solar field ♦ Existence of a regional center of excellence in electricity management ♦ High-level training framework on RE ♦ Existence of prepaid meters ♦ Increase performance in grid efficiency ♦ Increase in access, stability & security supply ♦ Increasing energy mix	♦ Ageing of transmission and distribution line ♦ Transmission and distribution lines losses ♦ Limitation in transmission and distribution systems ♦ Existing anarchic electricity connection ♦ Inexistence of Off-Grid code ♦ Need to improve the grid access ♦ Need to improve and update the system quality and security standards for transmission, distribution, and Off-Grid ♦ Inexistence of a program on the transfer of technology on RE ♦ Lack of cooperation between Universities and Ministries in charge of energy ♦ Weak collaboration between Ministries ♦ Lack of collaboration between Ministries and local authorities in the RE sub-sector ♦ Insufficient development of Public-Private Partnership in the RE sub-sector ♦ Inexistence of deconcentrated services in the energy sector ♦ Sustainability related issue ♦ Drop in grid reliability & energy efficiency ♦ Insufficient working capital
Legal	♦ Law on the electricity sector (2000) ♦ Law on electricity production from renewable energy sources (2018) ♦ Law on distribution governance in the electricity sector ♦ Off-Grid governance is stated in the Law on the promotion of electricity production from renewable energy sources ♦ Implementation text of the law on renewable energies ♦ Regulatory framework not very advantageous for small operators	♦ Lack of duty and tax relief on RE equipment importation of non-project need ♦ Poor regulatory framework for small operators
Environmental	♦ Good solar potential is favorable for the development of solar energy in all regions. ♦ Good hydro potential for micro and macro plants	♦ Weakness of the wind potential ♦ Lack of complete hydrological and meteorological data

**Table 4:** SWOT/PESTLE analysis of external factors outputs.

SWOT/PESTLE	External factors	
	Opportunities (+)	Threats (−)
Political	♦ Existence of a policy of achieving energy for all by 2030 in the country ♦ Agreement of the country through WAPP for integrated regional power markets across West Africa ♦ Electrification strategy policy ♦ Liberalization of electricity generation ♦ Off-Grid subsystem opened to private sector participation but may be improved ♦ Existence of a procurement process for power generation and regulation by the regulatory agency (ARSE) under the supervision of the Ministry of Energy ♦ Possible power generation and Off-Grid authorization and permits ♦ Generation Off-taking Options possible after an agreement ♦ Adoption of taxes reduction regarding RE equipment importation ♦ Ratification of international and regional agreements on RE and energy efficiency ♦ Grid network extension ♦ Aspirational target of RE in the energy mix	♦ Electricity sector is not liberalized ♦ Power sector framework under the State Monopoly ♦ Transmission and distribution are not opened to private-sector participation ♦ Transmission and distribution authorization and permit are not possible for now ♦ Power sector competition is under development for generation, is opened but tight in case of Off-Grid and not exist for transmission and distribution ♦ Extractive industry companies are not fairly treated in the supply of electricity by CEB and CEET as reported by ARSE ♦ Political instability ♦ Lack of continual and systematic incentives policies (Feed-In-Tarrif, tax preference, R&D subsidies, favorable financial interest rates, …) ♦ Political challenges faced by WAPP ♦ Lack of association of local Universities energy researchers in building, operation, and transfer (BOT) of power plants agreement.
Economic	♦ Growing economic development ♦ Regional power market financial benefits resulting from a low cost of operation and reduction of generation cost through WAPP ♦ Need for more investment in power generation, transmission and distribution ♦ Existence of economic regulation for transmission, distribution and Off-Grid (It may be improved for the latter) ♦ Water-Energy-Agriculture nexus opportunities ♦ Need of energy for agricultural products transformation (industry) ♦ Building of new industry platforms (PIA, etc …) ♦ Availability and willingness of technical and financial partners and financial partners for the development of RE ♦ Existence of related financing mechanisms with multilateral agreements; ♦ Existence of several donor initiatives in favor of the RE sector; ♦ Competitiveness of RE technologies and affordability;	♦ Inexistence of power generation economic regulation ♦ Inexistence of power system credit enhancement ♦ Difficult access conditions to financing ♦ Lack of funding to support the private sector ♦ Difficulty to access bank guaranties ♦ Economy threats such as the pandemic (COVID 19, etc.) ♦ Lack of effective trading institutions, and strong commercial arrangements among West African countries
Social	♦ 39.47 % of the population needs access to electricity, mostly in rural areas ♦ Need of potable water ♦ Need of water for irrigation at any period of the year ♦ People's Enthusiasm for RE ♦ Continual growth in demand for electricity ♦ Strong involvement of CSOs and the private sector in the development of RE ♦ Existence of local expertise ♦ Growth in demand for electricity, especially in rural areas rural areas ♦ Strong will of human development	♦ Insecurity (terrorists attacks in the North, .) ♦ Population's low purchasing power ♦ Power theft ♦ Pandemic (such as Ebola, COVID-19, etc …) ♦ Lack of effective strong collaboration among policymakers, regulators and utilities
Technical	♦ Possibility of cross-bordering power interconnection to provide cheaper, cleaner and more abundant energy resources to those in need ♦ Need of electricity network extension ♦ Possibility of bioenergy plant construction ♦ Ease of benchmarking in the field of RE ♦ Availability of the results of pilot projects on RE ♦ Many years of experience in power system implementation, operation and management by CEB, CEET, ARSE, AT2ER and Minister of Energy (MME) ♦ Rehabilitation of the underground distribution network mainly under PASET program ♦ State's aspirations for electricity security, reliability, and power grid resiliency ♦ Energy efficiency and energy conservation possibility	♦ Lack of meteorological data for a better energy planning and monitoring process ♦ Insufficient local expertise ♦ Insufficient organization of the actors ♦ Inexistence of ring lines at a high voltage of more than 20 kV around Lomé built to allow the exchange in the distribution network, in case of need ♦ Lack of effectiveness of the right infrastructure ♦ Inadequate maintenance of the grids
Legal	♦ Existence of a favorable framework for the creation of business (power generation and Off-Grid system)	♦ Electricity sector legal framework is not in harmony with the ECOWAS electricity market
Environmental	♦ Available lands and favorable conditions with less obstacles for electricity production, transmission, and distribution, and Off-Grid	♦ Possible conflict between arable land and areas allocated for renewable energy plants ♦ Climate change adverse impact on power systems ♦ Terrorism attacks ♦ Natural disasters ♦ Climate change extreme events (hot days, …)

### 3.3 Suggestions for a sustainable power system development

A sustainable electric power system is all about a system that can to supply secure and dependable power to consumers for the short and long run. Such a system must be put in place in any sovereign country. Towards that purpose, this section formulates suggestions for transforming the existing system. Power system transformation is motivated by the fact that governments as well as energy operators must address the urgent and pressing need for power at national, regional, and continental levels. Over time, this situation may remain unchanged. Because transforming the power system sustainably creates a conducive environment (policy and regulation) for building a more robust economic system through planning and best practices [70], there is need to do something about it. Therefore, synergy of actions is to be implemented to improve power sector governance, energy efficiency, competition and market, and power sustainability. This will radically make a contribution to power security [[60], [61], [62]]. Of course, an effective electric power system needs the right investment, compatible equipment, and technology, as well as the expertise to harvest existing energy resources like renewable throughout the country. These efforts will enable the community to produce power that is less harmful to the environment. In addition, the utility must safely dispatch this power to the population in a sustainable, affordable, secure, and reliable manner. The following is a tentative model that highlights the fundamentals of power systems that may improve the quality and reliability of the supply.

1) Desired future state of the State or the population

A prosperous economic future for the Togolese could be described or measured by the following factors:

♦ Improvement of the level of development in the country,
♦ Improvement in purchasing power,
♦ Improvement in the well-being and lifestyle of the population, and
♦ Decrease in political and socio-environmental issues.
♦ Continual industrialization and urbanization
♦ Modernizing agriculture across the country to increase productivity
♦ Agricultural products transformation in place

Any improvement in the quality of people's lives requires a lot of energy.

2) Strong policy and regulation

Energy policy and regulation are official documents written and promulgated by the government authority that present what is allowed and not, the objectives pursued, the orientations and visions, the administration of the sector. Hence, policy and regulation set the operatic conditions inside and outside the system. The current state of the power sector is hereby presented. Energy policy and regulation must be sturdy and benefit residents at all socioeconomic levels. A clear vision regarding power provision at temporal and spatial levels is developed to ensure the continuous supply of energy. Promotion of green economic and social development is ensured through, electricity production with respect to the environment. Also a set of rules for regulating power generation, transmission, and distribution in the country competitively and fairly is highlighted. These rules serve as tools for monitoring prices and standards. To follow and monitor power operations, the right procedures must be in place:

♦ Power standards,
♦ Grid code from generation to distribution,
♦ Grid access schemes,
♦ Grid efficiency and reliability,
♦ Off-grid standards,
♦ Feed-in tariff specification and conditions, and
♦ Market liberalization for fair competition.
♦ Power security

These sets of policies, standards, and best practices encourage and promote the use of decentralized power systems near the end-users.

3) Expansion of the installed capacity and planning

In an emerging country, enlarge installed power capacity and plan are crucial. The expansion of existing power capacity is closely dependent on electricity demand growth. This rise in demand is induced by economic prosperity, population growth, fast urbanization, and some increase in household net income. It has to be smart and take into account the time scale. The planning framework takes into account various factors [70] including the 1) interplay of trends in the power system, 2) interplay between bulk-system or base system, 3) distributed and possible future demand-side resources, and 4) strategies of addressing reliability and flexibility measures. Additionally, the planning framework will continue to support and promote resource conservation and emissions reduction over the years. Therefore, utility in charge of the subsector monitors the system to make sure that standards and policies are met.

**Fig. 10:**
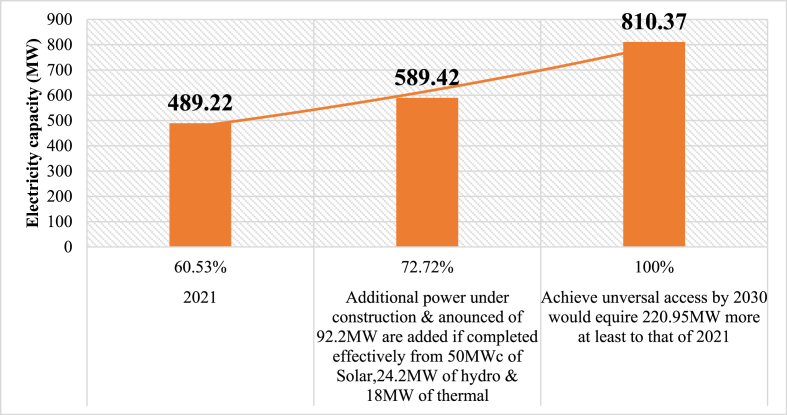
Electricity capacity left to achieve universal access to energy in 2030 (100 %) considering 2021 space of power demand.

In order to keep the pace of progress, the current effort of the State has to be maintained and sustained. As of 2021, the installed power capacity and the importation accounted for 489.22 MW. Based on that capacity, the utility provided electricity to up to 60.37 % of the population. With the ongoing plants' construction and announced, a large part of the rest of the population that lacks electricity, could rejoice to be granted access. The Kekeli Efficient plant if completed and in operation will provide an additional 18 MW, AMEA solar plant with an additional 20 MWc, 24.2 MW from the complete construction and operation of Sarakawa hydropower and 30 MWc from the construction and operation of Dapaong solar plant. Therefore, an additional power of 92.2 MW would be added to the available capacity in 2021 giving a capacity of 589.42 MW. Consequently, an equivalent access rate to the electricity of 72.73 % would be reached if everything is equal (Fig. 10). So, under the present days' socioeconomic conditions, for the country to achieve universal access to energy by 2030 (100 %) as planned in the electricity strategy, it would require an additional effective capacity of 220.95 MW at least, giving a total demand of 810.37 MW. This projection is consistent with ECOWAS’. The regional Organization projected that the Togolese demand peak could reach 821 MW in 2033 [48].

**Fig. 11:**
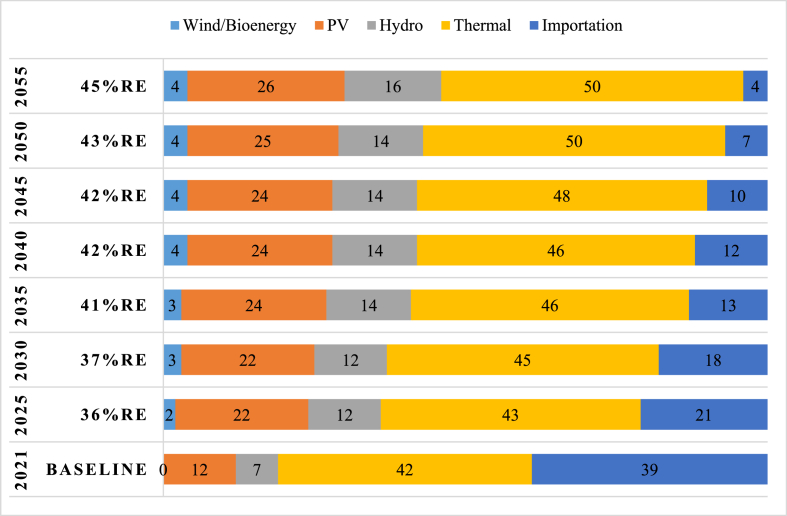
Energy mix generation plan.4)Transmission transformation

Considering the baseline of 2021, a prospect of energy mix planning is proposed and presented in Fig. 11. It is a tentative plan to shed light on the possible extent to which sustaining the ongoing State effort could take the country. During the previous ten years, Togolese State realized the implementation of 165 MW of thermal (100 MW from CONTOUR GLOBAL TOGO and 65 MW from KEKELI EFFICIENT POWER SA out of which 18 MW remained to be completed at the end of 2021). On the contrary, regarding RE, a capacity of 58.12 MWc of solar was installed while 24.2 MW of hydro and 30 MWc of solar were announced to be implemented shortly. Based on the present trend, the growth in electricity production capacity would be in a decade to come such that solar PV system installed capacity would increase faster than the one of hydro. Thermal installed capacity as far as it concerned will still take the lead for the good reason it will continue to guarantee the baseload for the country. With the completion of the ongoing regional grid integration project, the cost of grid-connected PV systems' initial investment could be reduced since storage energy systems may not need anymore. RE in Togo or any other country of the region will be injected into the regional network and this could lead to increased production and the mitigation instability of variable RE.

The electricity transmission subsystem comes after electricity generation subsystem. It has to deal with transporting power at high voltage to avoid loss to distribution station. The transformation of this system is more than important in order not only to update the system but also to reduce the distortion in voltage and current and/or frequency deviation that likely causes the malfunction of equipment [71]. More, having this transformation would result in quality and reliability improvement as well as in reducing power losses [[72], [73], [74]]. Following that, a plan needs to be developed according to the supply and its growth, the urbanization and the overall development of the nation. Thus, a monitoring system has to be settled and operated actively to identify and replace overused equipment. The following are required as seen in Table 5. A transmission system that is transformed to meet the requirements could provide consumers with a secure, reliable and continual flow of power.

5) Distribution transformation

**Table 5:** Transmission transformation [75].6)Effective and active collaboration with other countries in West Africa

Components of transformed Transmission	Sub-components of transformed Transmission	Description
Control centers smarter	Transmission system monitoring	Data Collection based on SCADA and Phasor Measurement Units (PMUs) [76] coupled with wide-area geographical information (GIS)
	Analytical capability	Provision and installation control centers capable of performing dynamic model updates and validation from cascading failure [77]
	Controllability	Possibility to conduct real-time studies in place of offline studies for proactive and adaptive actions to better combine generation-transmission
	Interactions with the electricity market	The electricity market is included in power systems and designed and regulated to meet efficient electricity standards
Transmission networks smarter	Ensure high efficiency and quality in the transmission networks by using high-capacity AC and DC facilities	Usage of advanced power electronics such as FACTs and HVDC) to ensure flexible controllability and transmission reliability improvement. Usage of solid-state transformers and solid-state breakers in place of traditional electromagnetic transformers and mechanical breakers.
	Ensure robust and self-healing and transmission	Achieved by using advanced sensing and monitoring of breakers and the transformers
	Advanced transmission facility maintenance	Possibility to lubricate the moving parts
	Extreme event facility hardening system	Possibility to prevent extreme events
Substations smarter	Digitalization platform	Fast and reliable sensing, measurement, communication, protection and maintenance of equipment and apparatus installed possible
	Autonomy	Each substation is independent but interconnected with others
	Coordination	Easy communication with other substations and control centers
	Self-healing	Substation reconfigures itself dynamically

The distribution subsystem follows the transmission subsystem in an electric power system. This subsystem is responsible for furnishing electricity to consumers at low voltage. Therefore, it also needs to be transformed and planned to facilitate electricity access and satisfy the need of the population. To achieve that, the expansion of the distribution lines plan has to be maintained and sustained according to the rate of urbanization and the growth in the income of the residents. Furthermore, the advanced metering infrastructure (AMI) technology has to be explored and implemented; and an upgrading of the entire subsystem is undertaken to reduce the lines losses and increase the quality of the supply.

Entertaining a project like a West Africa Power Pool (WAPP) to establish a regional power market among the 14 countries in the region is promising. WAPP created by decision A/DEC.5/12/99 of the ECOWAS Authority of Heads of State of Government envisions to combine country wide electricity systems into a unified regional electrical energy market with the ultimate intention of supplying in the medium and long term, a regular and dependable electricity at competitive cost to the population of the region. Coming and working together as countries will not be without challenges However, overcoming those challenges is vital to build such a great project which would result in priceless benefits for all [78]. Thus, an effective regional power interconnection may provide solutions for interrelated power issues the region is facing such as limited access to electricity, electricity security and reliability-related issues and ageing of the power infrastructures. Indubitably, electricity grids integration would favor sustainability in power systems by improving:

♦ Electricity reliability.
♦ Electricity affordability by allowing countries that come together to benefit from low costly resources granted in the region.
♦ Sustainability of power generation and supply by displacing baseload oil-fueled power generation with cleaner sources of electricity such as natural gas, solar, hydropower, …
♦ Resiliency of power system through ease balance of energy storages and therefore mitigation of the intermittency of RE.
♦ Private sector investment attraction in power production.

## 4 Conclusions

This study investigated ways to improve the power systems of emerging economies. To achieve the objectives of the study, a thorough literature survey/review and interviews of energy experts have been utilized to assess the existing Togolese power system. A SWOT/PESTLE scheme has been then applied to analyze in depth and all-encompassing the power system to bring out internal (strengths and weaknesses) and external (opportunities and threats) factors impacting it. The following parameters have been considered 1) politics (decisions), economics (profits), social (people), technology (knowledge), and environment (planet). Furthermore, suggestions were made to transform not only the systems but also to improve the related reliability and resiliency indices. The findings revealed the following:

❖ The national power generated and the importation accounted for 489.22 MW that supplied 60.37 % of the population broken down as follow: 19.50 % of rural and 94.10 % of urban areas. Thus, the country would require a minimal assured supply of 220.95 MW in addition to that of 2021 to achieve 100 % electrification by 2030 provided by its energy mix resource (hydro, solar, bioenergy, thermal, …).
❖ The interruption regardless of the origin lasted for a minimal of 2 h (CAIDI>2) and the losses in the distribution network accounted on average for 16 % from 2017 to 2021 (while the standard loss admitted is 10 %). The electricity access poverty accounted for 80.50 % in rural, 5.90 % in urban and 39.47 % at countrywide level.
❖ A number of deficiencies and problems hamper the electricity sector such as heavy dependence on electricity importation, limited production, aging of infrastructures, policy and regulation-related issues, limited coverage of the national grid, limited investment, incentives and power rebate-related issues, and grid code and access issues.

Solutions have to be provided to these issues to improve the power systems. Accordingly, the oriented-based model for power resiliency was proposed as a temporary solution. A solution-based model could enable the entire power system to be transformed into a conducive environment with a solid power management system and a realistic investment plan. Such transformation may be possible through innovating the transmission and distribution networks as well as enforcing the effectiveness of regional collaboration. Thus, a continual improvement of the electric power system would guarantee the reliability of the electricity supply in Togo and West-Africa at large.

The study has not addressed power system management in emerging economies in details. Therefore, for future studies, it is suggested to investigate electricity management as well as the new power reliability concept described as Customer Enjoy Without Interruption Index (CEWII) in these countries.

## Data availability statement

The data used in the study will be provided upon request.

## CRediT authorship contribution statement

**Kokou Amega:** Writing – original draft, Visualization, Validation, Software, Resources, Methodology, Investigation, Formal analysis, Data curation, Conceptualization. **Yacouba Moumouni:** Writing – review & editing, Visualization, Validation, Methodology, Conceptualization. **Yendoubé Laré:** Writing – original draft, Supervision, Resources, Funding acquisition. **Ramchandra Bhandari:** Writing – review & editing, Supervision, Resources, Methodology, Formal analysis. **Pidename Takouda:** Validation, Resources, Data curation. **Saidou Madougou:** Supervision, Project administration, Conceptualization.

## Declaration of competing interest

The authors declare that they have no known competing financial interests or personal relationships that could have appeared to influence the work reported in this paper.
